# A practical guide to pre‐trial simulations for Bayesian adaptive trials using SAS and BUGS

**DOI:** 10.1002/pst.1897

**Published:** 2018-09-14

**Authors:** Christian Holm Hansen, Pamela Warner, Allan Walker, Richard A. Parker, Lucy Whitaker, Hilary O.D. Critchley, Christopher J. Weir

**Affiliations:** ^1^ MRC Tropical Epidemiology Group London School of Hygiene and Tropical Medicine London UK; ^2^ Usher Institute of Population Health Sciences and Informatics University of Edinburgh Edinburgh UK; ^3^ Edinburgh Clinical Trials Unit University of Edinburgh Edinburgh UK; ^4^ MRC Centre for Reproductive Health University of Edinburgh Edinburgh UK

**Keywords:** adaptive trials, Bayesian modelling, OpenBUGS, SAS, simulations, WinBUGS

## Abstract

It is often unclear what specific adaptive trial design features lead to an efficient design which is also feasible to implement. Before deciding on a particular design, it is generally advisable to carry out a simulation study to characterise the properties of candidate designs under a range of plausible assumptions. The implementation of such pre‐trial simulation studies presents many challenges and requires considerable statistical programming effort and time. Despite the scale and complexity, there is little existing literature to guide the implementation of such projects using commonly available software. This Teacher's Corner article provides a practical step‐by‐step guide to implementing such simulation studies including how to specify and fit a Bayesian model in WinBUGS or OpenBUGS using SAS, and how results from the Bayesian analysis may be pulled back into SAS and used for adaptation of allocation probabilities before simulating subsequent stages of the trial. The interface between the two software platforms is described in detail along with useful tips and tricks. A key strength of our approach is that the entire exercise can be defined and controlled from within a single SAS program.

## 1 INTRODUCTION

There has been growing realisation among statisticians that adaptive trial designs can improve the efficiency of drug development and therefore offer advantages over conventional trial designs. As a result, Bayesian adaptive designs have become increasingly common especially in early‐phase research such as dose‐finding studies which benefit from the additional flexibility offered by Bayesian modelling techniques.^1^ There are numerous ways of designing an adaptive trial, so how do you decide how many adaptations to build in, when to adapt, what adaptation rules to implement, and so on? The task of identifying an optimum adaptive trial design is not straightforward and usually pre‐trial simulations are required to evaluate the properties of candidate designs.

For many clinical trial statisticians, the SAS software is their main statistical programming platform, but while some Bayesian modelling needs can be addressed using Proc MCMC in SAS, the Bayesian inference Using Gibbs Sampling (BUGS) software offers a highly flexible and intuitive environment for specifying and fitting Bayesian models of almost any level of complexity. There are two main versions of the BUGS software: OpenBUGS is an open‐source version which is continually being developed, while WinBUGS, which was developed first, is a stand‐alone, stable and well‐established version of the software (according to OpenBUGS' own website) which will remain available although will not be further developed.^2^, ^3^, ^4^ Zhang et al (2008) published a useful tutorial with details of a SAS interface for Bayesian analysis with WinBUGS.^5^ In this Teacher's Corner article, we extend this idea, illustrating in a step‐by‐step guide how SAS and the BUGS software may be used to coordinate simulations of Bayesian adaptive trials, pointing out differences in the implementation with WinBUGS and OpenBUGS where necessary.^6^

## 2 MOTIVATING EXAMPLE

Firstly, we introduce as a motivating example the response‐adaptive dose‐finding DexFEM trial (Dexamethasone For Excessive Menstruation study, funded by the MRC, ref: MR/J003611/1). This trial aims to establish whether Dexamethasone (a synthetic glucocorticoid) taken orally is effective in reducing menstrual blood loss in women with heavy menstrual bleeding and to estimate the Dexamethasone dose‐response profile.^7^ The trial aims to enrol 108 women with heavy menstrual bleeding (expected to lead to 100 being available for the final analysis after allowing for drop‐outs) in a multiple parallel arm dose‐finding trial, with differing dose levels.

Participants are randomised to receive either placebo or one of a range of possible Dexamethasone doses with, initially, equal allocation probabilities across the active doses. In a response‐adaptive trial such as DexFEM, efficient learning around the critical region of the underlying dose‐response curve (ie, the region that covers the shift between efficacious or not) can be achieved by updating the allocation probabilities for active doses for subsequent trial participants as outcome data from participants already in the trial provide information on the likely shape of the dose‐response curve.

There are many design features to consider when planning a response‐adaptive dose‐finding trial. Those considered for the DexFEM trial included the number of doses to be studied (and therefore the number of trial arms to include), how many adaptations to build into the trial, and when during the trial to perform those adaptations. The study team sought a feasible design which would provide effective adaptation and therefore carried out a simulation study to characterise the performance of a range of candidate designs in terms of type I error and statistical power.

### 2.1 Simulation of trial data

The simulations aimed to mimic a real trial as closely as possible, including the screening and enrolment process, randomisation to trial arms, and treatment effects of receiving the trial drug at various doses.

The primary outcome variable is the difference between the baseline and follow‐up menstrual blood loss measurements. To be included in the trial, participants have to have an average blood loss of at least 50 mL over two screening cycle menstrual blood loss measurements. Correlated measurements on the same woman were simulated, and because only subjects with high baseline measurements would be eligible for study participation, the simulation procedure induced a “regression to the mean” effect in the follow‐up measurements as might be expected in a real trial.

After generating baseline measurements at screening, simulated patients with a baseline average blood loss of at least 50 mL were randomised to either placebo or an active dose according to the current set of randomisation probabilities. After randomisation, the next step was to simulate measurements during treatment after the addition of a treatment effect, governed by a hypothesised dose‐response relationship.

### 2.2 The Bayesian trial analysis

The dose‐response curve was estimated from the simulated trial data using a Bayesian second‐order Normal Dynamic Linear Model.^8^ Such flexible models place few restrictions on the shape of the estimated curve. The freely available BUGS software was used to fit the Bayesian model with the simulated data being generated using SAS.

With this motivating example in mind, we provide in the following sections the framework for the dialogue between SAS and the BUGS software (highlighting minor differences between WinBUGS and OpenBUGS) and illustrate how to fully automate and manage the simulation exercise using a single SAS control program.

## 3 HOW TO RUN BUGS THROUGH SAS

Fitting a Bayesian model using BUGS requires a data file, a model specification, and a set of initial values to start off the Markov Chain Monte Carlo (MCMC) iterative algorithm.^9^ In addition, because the simulations are coordinated within SAS, the interactive BUGS user interface is not being used. A so‐called script file is therefore also required. This is a file that contains all the BUGS instructions needed to specify the model, estimate posterior distributions of the model parameters, and export realisations and summaries from these.

The simulated trial data required for the Bayesian analysis are stored in a single SAS dataset and exported from SAS as a text file in a data format accepted by BUGS to a location on a hard drive or server. A freely accessible SAS macro is available for download (http://www.psychstat.org/us/article.php/61.htm) and allows this data export and manipulation exercise to be performed in one simple step through a single macro call.^10^, ^11^ To ensure that the macro can be accessed automatically each time SAS is opened, save the downloaded files in the system directories where SAS searches for macro programs by default (eg, core\sasmacro), or add the path of the directory containing the macro files to the SASAUTOS clause of the SAS configuration file.

Next, we use SAS to generate a model specification file that specifies the Bayesian model to be estimated in BUGS. This is done by including the model specification in a SAS dataset using the SAS CARDS command and outputting this as a text file to the external location on the hard drive or server. The SAS data steps needed to perform these tasks are shown in Box 1.

**Box 1 SAS data steps needed to create and export the model specification file which contains the Bayesian model to be estimated in BUGS. The first data step creates a SAS dataset, MODEL, comprising a single cell which contains the entire model specification. In the subsequent _NULL_ data step, the content of the MODEL dataset is stored as a text file.**

DATA MODEL;

INPUT model $80.;

CARDS;

model{

<Include BUGS model and prior specifications here>

}

;

RUN;

DATA _NULL_;

SET MODEL;

FILE "<file path>\BUGS\Model\Model.txt";

PUT model;

RUN;

Similarly, a SAS dataset containing text specifying initial values for all of the model parameters is created and saved as a text file (Box 2). Finally, the text for the BUGS script is specified in a SAS dataset and saved as a text file in the folder containing the BUGS executable file. Also saved to this location is a Windows batch file with a command to execute BUGS using the script file (Box 3).

**Box 2 Text for the BUGS initial values specification is stored in the SAS dataset INITs and subsequently saved in a text file.**

DATA INIT;

INPUT initials $80.;

CARDS;

list(<parameter1=parameter1initialvalue, parameter2=parameter2initialvalue, etc.>

<Specify further initial parameter values here>

)

;

RUN;

DATA _NULL_;

SET INIT;

FILE "<file path>\BUGS\InitialVals\IN1.txt";

PUT initials;

RUN;

The script file contains commands to open up a BUGS log window, check model specifications, load data and compile the model, and set initial values. Depending on the particular model being fitted it may be necessary to generate additional initial values not included in the initial values specification, eg, to model incomplete data or predictive distributions. This can be done with WinBUGS by adding the *gen.inits* command (OpenBUGS: *modelGenInits*) after reading in the initial values specification. The *update* command (OpenBUGS: *modelUpdate*) is used on two occasions, firstly to update the sampler a sufficiently large number of times (in a so‐called “burn‐in” phase) to obtain convergence in the MCMC algorithms, and secondly, to draw further realisations for inference after convergence has been achieved. In our example, after a “burn‐in” phase of 5000 realisations, a further sample of 10,000 realisations of a set of relevant parameters is recorded and summary statistics are computed and written to the log file. See Box 3a and 3b for example code to use with WinBUGS and OpenBUGS, respectively.

The last step required to start the BUGS analysis from SAS is a call to execute the batch file. This is easily done using the SAS X command:

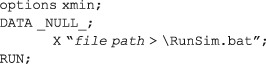

**Box 3a SAS data steps needed to generate and save the WinBUGS script and Windows batch files. In the first data step a SAS dataset, SCRIPT, is created which contains the WinBUGS script. The subsequent _NULL_ data step then stores the script in a text file named simbat.txt. The dataset BATCH contains the batch instructions to execute WinBUGS using the script stored in simbat.txt. The final _NULL_ data step stores these instructions in a batch file named RunSim.bat in the same directory as the WinBUGS executable file.**

DATA SCRIPT;

SET SEED(where=(<set condition for selecting a new seed from dataset of seeds,

e.g,. trial=1 AND phase=2>));

LENGTH script $80.;

SCRIPT = "display('log')" ;OUTPUT;

SCRIPT = "check('<file path>/BUGS/Model/model.txt')" ;OUTPUT;

SCRIPT = "set.seed("||catt(WINSEED)||")" ;OUTPUT;

SCRIPT = "data('<file path>/BUGS/Data/dat_sim.txt')" ;OUTPUT;

SCRIPT = "compile(1)" ;OUTPUT;

SCRIPT = "inits(1, '<file path>/BUGS/InitialVals/in1.txt')" ;OUTPUT;

SCRIPT = "update(<number of burn‐in iterations, e.g,. 5,000>)" ;OUTPUT;

SCRIPT = "set(<name of 1st parameter>)" ;OUTPUT;

SCRIPT = "set(<name of 2nd parameter>)" ;OUTPUT;

<etc>

SCRIPT = "update(<number of monitored iterations, e.g,. 10,000>)" ;OUTPUT;

SCRIPT = "stats(*)" ;OUTPUT;

SCRIPT = "save(<file path>/BUGS/output/log.txt')" ;OUTPUT;

SCRIPT = "quit()" ;OUTPUT;

RUN;

DATA _NULL_;

SET SCRIPT;

FILE "C:\Program Files\WinBUGS14\simbat.txt";

PUT script;

RUN;

DATA BATCH;

INPUT batch $80.;

CARDS;

C:

CD C:\Program files\WinBUGS14

WinBUGS14.exe /PAR simbat.txt

EXIT

;

RUN;

DATA _NULL_;

SET BATCH;

FILE "C:\Program Files\WinBUGS14\RunSim.bat";

PUT batch;

RUN;

Control is returned to SAS once the RunSim.bat Windows batch file has finished executing. Be aware that if the file path takes the form *C:*\*Program Files*\*..*., the SAS X command may not recognise the *Program Files* directory unless this is referred to by its 8‐character system name instead (usually something like *C:*\*Progra~1*\ *…*).The *xmin* option is used to suppress the WinBUGS application window by starting the program in minimised form. This is particularly useful when running the SAS program code in batch mode, as the computer may be used for other tasks while SAS is simulating data and running WinBUGS in the background. The *xmin* option only works with WinBUGS; however, the HEADLESS option used with the OpenBUGS application call (Box 3b) serves the same function.

**Box 3b SAS data steps needed to generate and save the OpenBUGS script and Windows batch files. The function of each data step is described in the explanatory text accompanying Box 3a. For the OpenBUGS application call to work properly, we found it necessary to include a blank space at the end (forced by the trailing “#”) of the line and to restrict the name of the script file to six characters in length. The reasons for this are unknown to us.**

DATA SCRIPT;

SET SEED(where=(<set condition for selecting a new preset state for OpenBUGS, random number generator, e.g,. trial=1 AND phase=2>));

LENGTH script $80.;

SCRIPT = "modelOutput('log')" ;OUTPUT;

SCRIPT = "modelCheck('<file path>/BUGS/Model/model.txt')" ;OUTPUT;

SCRIPT = "modelSetRN("||catt(RNstate)||")" ;OUTPUT;

SCRIPT = "modelData('<file path>/BUGS/Data/dat_sim.txt')" ;OUTPUT;

SCRIPT = "modelCompile(1)" ;OUTPUT;

SCRIPT = "modelInits(1, '<file path>/BUGS/InitialVals/in1.txt')" ;OUTPUT;

SCRIPT = "modelUpdate(<number of burn‐in iterations, e.g,. 5,000>)" ;OUTPUT;

SCRIPT = "samplesSet(<name of 1st parameter>)" ;OUTPUT;

SCRIPT = "samplesSet(<name of 2nd parameter>)" ;OUTPUT;

<etc>

SCRIPT = "modelUpdate(<number of monitored iterations, e.g,. 10,000>)" ;OUTPUT;

SCRIPT = "samplesStats(‘*’)" ;OUTPUT;

SCRIPT = "modelSaveLog(<file path>/BUGS/output/log.txt')" ;OUTPUT;

SCRIPT = "modelQuit(‘yes’)" ;OUTPUT;

RUN;

DATA _NULL_;

SET SCRIPT;

FILE "C:\Program Files\OpenBUGS\OpenBUGS323\simbat.txt";

PUT script;

RUN;

DATA BATCH;

INPUT batch $80.;

CARDS;

C:

CD C:\Program files\OpenBUGS\OpenBUGS323

OpenBUGS.exe /HEADLESS /PAR simbat.txt #

EXIT

;

RUN;

DATA _NULL_;

SET BATCH;

FILE "C:\Program Files\OpenBUGS\OpenBUGS323\RunSim.bat";

PUT batch;

RUN;

USEFUL TIP

It is helpful to direct the SAS procedure output and log to text files on the hard drive or server as these will otherwise fill up and need to be emptied regularly. The two commands.

 PROC PRINTTO print = “<*file path* > \SAS\Output\sim001_01.lst” new;

 RUN;

and

 PROC PRINTTO log = “<*file path* > \SAS\Log\sim001_01.txt” new;

 RUN;

are used to direct the SAS output and log to external files respectively. Note that it is often convenient to store the first part of the file path in a macro variable which can be defined at the top of the program.

### 3.1 Controlling the seed for WinBUGS' random number generator

You may be wondering why in Box 3a and 3b the script file is generated as a sequence of string values. You can use the CARDS command instead if you prefer, except that it is then not possible to vary the random number seed in successive WinBUGS or OpenBUGS calls. The Gibbs sampler in BUGS uses pseudorandom numbers to generate realisations from posterior distributions of estimated parameters, but unless explicitly set, the seed for the random number generator always reverts to its default value each time BUGS is executed. To change the random number seed in WinBUGS, use the *set.seed()* command in the script file with the new seed included within the parentheses. This can be done by updating the script file with a new seed from a list of seeds that has been generated a priori and stored in a dataset as shown in Box 3a where the SEED dataset is used in the data step that generates the script file and the relevant seed (stored in the WINSEED variable) is picked out. With OpenBUGS, the user has the option instead to change the internal state of the random number generator to one of 14 predefined states. This can be done through a script file using the command *modelSetRN (x)*, where *x* is an integer from 1 to 14.

### 3.2 Debugging of the BUGS program and checking for convergence of the MCMC chain

While it is possible to relay error messages generated in BUGS back to SAS, we would not recommend using this approach for de‐bugging when developing the Bayesian model as this would add an unnecessary layer of complication. Instead, we would advise working directly in BUGS when first specifying the Bayesian model, and ensuring at that stage that any problems are addressed with respect to model specifications, initial values, script files etc. We would only recommend turning to SAS to automate multiple runs of the Bayesian model after the model has been developed satisfactorily within BUGS's graphical user interface. There is then no need to facilitate the flow of error messages from BUGS to SAS.

It is unlikely to be feasible to use SAS to assess convergence of every MCMC chain because from each of possibly millions of chains this would require thousands of realisations to be passed to SAS for evaluation and computation of model diagnostics. Computationally, this would be extremely intensive, and even if feasible, it would not be humanly possible to assess each chain for convergence. Instead, we would advise that convergence is considered qualitatively during model specification, and by implementing spot checks for selected design scenarios and hypothesised dose‐response relationships, at which points it is feasible to carefully inspect properties of trace plots and posterior distributions and convergence of multiple chains with over‐dispersed initial values, for example using the in‐built Brooks‐Gelman‐Rubin convergence diagnostic.^12^

### 3.3 How to import BUGS model parameter estimates into SAS

Once the Bayesian model has been fitted, posterior summary estimates of relevant parameters which have been saved by BUGS in a log file on the hard drive or server will need to be imported back into SAS. To do so, it is necessary to examine the log file carefully to ensure that it is deciphered correctly with parameter estimates being stored in the appropriate rows and columns. The data step in Box 4 is an example of how parameter estimates from a BUGS log file may be imported into a SAS dataset. Each line of the log file is read into a string variable and tab spaces are replaced with a single “X” using the *translate* function. Only lines pertaining to the relevant variables are retained before finally extracting the summary statistics and storing them as numeric variables.

**Box 4 Importing parameter estimates from a BUGS log file into a SAS dataset.**

DATA LOG;

INFILE "< file path >\BUGS\Output\log.txt" TRUNCOVER;

INPUT log $80.;

log=TRANSLATE(log,"X","09"x);

IF (SUBSTR(log, 2, <length of 1st parameter name>) EQ '< name of 1st parameter >')

OR (SUBSTR(log, 2, <length of 2nd parameter name>) EQ '< name of 2nd parameter >')

<etc>

;

parameter = PUT (scan(log, 1,'X'), $10.);

mean = INPUT (scan(log, 2,'X'), best12.);

SD = INPUT (scan(log, 3,'X'), best12.);

MC_error = INPUT (scan(log, 4,'X'), best12.);

P2_50 = INPUT (scan(log, 5,'X'), best12.);

median =INPUT (scan(log, 6,'X'), best12.);

P97_5 = INPUT (scan(log, 7,'X'), best12.);

RUN;

### 3.4 How to export realisations from Bayesian posterior distributions from BUGS to SAS

To evaluate the functions that governed adaptation in the DexFEM trial, it was necessary to perform calculations in SAS on the raw simulated data from BUGS. To export realisations from posterior distributions, simply include *coda* command lines in the WinBUGS script file (OpenBUGS: *samplesCoda*) for each parameter to be exported:

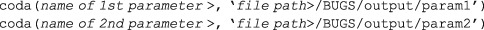

These lines will have to be inserted after setting the monitor for the relevant parameters with the *set* command (OpenBUGS: *samplesSet*) and running an adequate number of updates. We have included the SAS macro *CodaTransform* in Box 5 for reading the data from the coda files back into SAS and transforming them into a usable SAS dataset.

**Box 5 The CodaTransform macro for importing data from OpenBUGS coda files and transforming them into usable SAS datasets. To use with WinBUGS, change the file names in the first and second Proc Import call to *&Namepara.Index.txt* and *&Namepara..txt* respectively in order to match the slightly different coda file naming rules used by this version of the software. The macro has the following arguments: *Namepara* = <The parameter name specified in the "coda" command and included in the filename>; *Dim* = <The dimension of the parameter such as the number of trial arms, e.g. "7">.**

%MACRO CODATRANSFORM(Namepara=, Dim=);

PROC IMPORT datafile="<file path>/BUGS/output/&Namepara.CODAindex.txt"

out=&Namepara.Index0 REPLACE;

getnames=no;

RUN;

PROC IMPORT datafile="<file path>/BUGS/output/&Namepara.CODAchain1.txt"

out=&Namepara.chain0 REPLACE;

getnames=no;

RUN;

DATA &Namepara.Index;

SET &Namepara.Index0;

ITERATIONS = VAR3‐VAR2 + 1;

DO t = 1 TO ITERATIONS;

OUTPUT;

END;

KEEP VAR1 t;

RUN;

DATA &Namepara;

MERGE &Namepara.Index

&Namepara.chain0(DROP=VAR1);

RUN;

%IF &Dim.>1 %THEN %DO;

DATA &Namepara._final;

MERGE

&Namepara.(WHERE=(VAR1="&Namepara.[1]") rename=(VAR2="&Namepara.1"n))

%DO J=2 %TO &Dim.;

&Namepara.(WHERE=(VAR1="&Namepara.[&J.]")

rename=(VAR2="&Namepara.&J."n))

%END; ;

DROP VAR1;

RUN;

%END;

%IF &Dim.=1 %THEN %DO;

DATA &Namepara._final;

SET &Namepara.;

WHERE VAR1="&Namepara.";

RENAME VAR2="&Namepara."n;

DROP VAR1;

RUN;

%END;

%MEND CODATRANSFORM;

### 3.5 How to simulate adaptation

Model estimates from interim analyses are used to update the randomisation probabilities for subsequent participants enrolling during the next phase of the trial. It is good practice to store intermediate results such as the modelled parameter estimates and the set of new allocation probabilities after each interim analysis since these can be used to monitor the process of adaptation and may even be needed at a later stage for reanalysis to address questions unforeseen during the design development. Such results are easily added to a dataset by using the APPEND command of the SAS Datasets procedure.

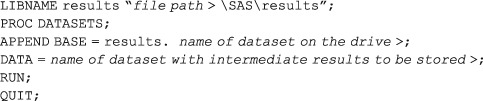

When saving such parameters, make sure to include a variable in the stored datasets which will allow identification of the particular trial run and adaptation stage which generated the data.

Once the allocation probabilities have been adapted, the trial progresses to the next phase by repeating the process of simulating baseline values at screening, randomising eligible subjects, inducing treatment effects according to the hypothesised dose‐response curve, and simulating outcome values at follow‐up. This goes on until the point of the next interim analysis when available data from all enrolled participants are passed to BUGS for model re‐estimation and estimates are fed back again to SAS for evaluation of the second adaptation, and so on. At the end of the simulated trial, the model is fitted one last time, and final treatment effect estimates are evaluated and stored. In order to determine design properties such as trial power and type I error rate with good precision, the whole trial simulation exercise is repeated many times.

### 3.6 A simple way to coordinate a complex simulation exercise

**Figure 1:**
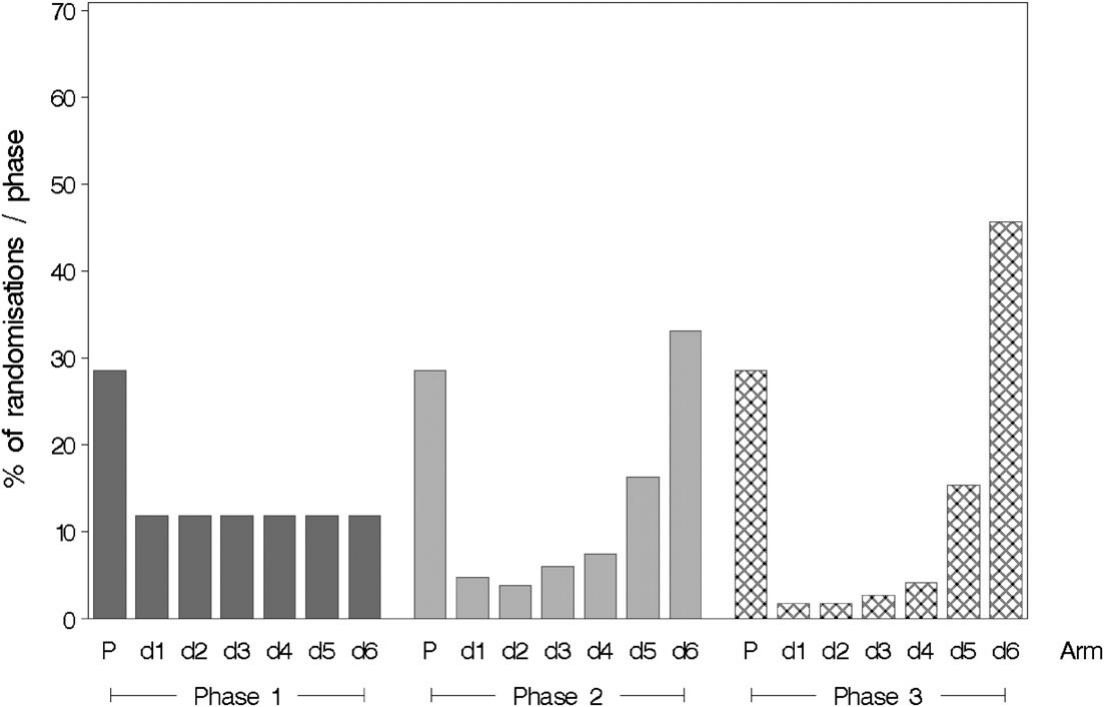
Allocation probabilities at each phase of a simulated dose‐finding trial with seven parallel trial arms (placebo (P) and six active dose levels (d1‐d6)) and two adaptations. The placebo allocation rate was held constant by design while allocation probabilities to the other arms changed following each adaptation interim analysis. The increase in allocation probabilities to higher dose‐levels during phase 2 and 3 reflects information gain over the course of the trial about the underlying dose‐response curve (which for the scenario in question was flat until dose level four)

By placing all core SAS programming code in SAS macros and using macro variables to denote key design parameters, it is possible to specify the entire set of trial scenarios to be investigated in a single control program. For example, in the DexFEM simulation study, the following macro call.

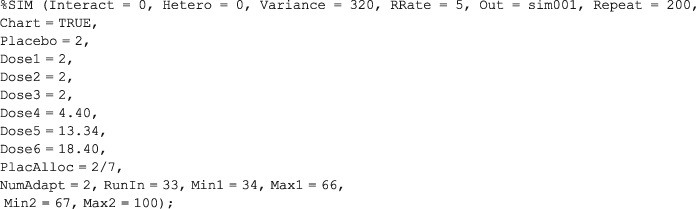

invoked a run of 200 trial simulations under a scenario with a recruitment rate of five patients per month, *
Recruitment rate can be important since a fast enrolment rate relative to the length of follow‐up may reduce the benefits of using an adaptive design.^12^
 a hypothesised dose‐response curve defined by *Placebo, Dose1,..., Dose6* (notice that a non‐zero effect has been placed on *Placebo* to emulate a placebo effect), a variance parameter of 320 without heteroscedasticity (*Hetero = 0*), and no baseline value‐by‐treatment interaction effect (*Interact = 0*). The design specified is a seven‐arm adaptive trial with two adaptations (one each after 33 and 66 patient randomisations), a placebo allocation rate of 2/7, and a total sample size of 100 participants. The *Out* variable ensures that all data files generated from these simulations have a unique identifying prefix. The *Chart = TRUE* option was used to generate a figure illustrating the way in which allocation probabilities changed through the different phases of the adaptive trial (Figure 1).

Finally, the SAS control program that contains the macro calls with details of every combination to be investigated can be executed in batch mode with a single program call. As well as being faster, batch mode execution allows the user to undertake other tasks on the computer while the simulations run their course. It is worth noting that significant speed gains may be achieved by directing file output to the local hard drive on the computer which is running the simulations instead of saving continually to a network server, although it is advisable to build in occasional back‐ups to the server to avoid loss of data in case files on the local PC become corrupted.

The findings from the DexFEM pre‐trial simulation study have been reported,^13^ and the trial design selected commenced recruitment in January 2014.^7^

## 4 DISCUSSION

In this Teacher's Corner article, we have illustrated how to carry out pre‐trial simulation work for a Bayesian response‐adaptive trial using SAS and WinBUGS or OpenBUGS, and in particular how to manage the dialogue between the two software platforms. A key strength of the approach is that the entire simulation task can be defined and controlled from within a single batch executable SAS program. We have included complete worked examples of such a program, one using WinBUGS and one using OpenBUGS, in the online supplementary appendix.

The simulations for the DexFEM study involved 200 repetitions each of about 150 different trial scenarios each requiring multiple adaptations and subsequent re‐estimation for every model parameter, resulting in a total of over one million MCMC runs which took approximately 6 weeks to run on a standard specification office PC. †
4‐core CPU with a clock speed of 3.30 GHz and 8 MB SmartCache. (Although this was the computational time needed for the simulations to complete, the task of designing and programming the simulation study took much longer.) We ran the simulations on two computers in parallel to speed up the process. Undoubtedly, the simulation time could have been reduced by programming bespoke routines, for example using C++ or a similarly low‐level programming language; however, this would have required additional skills and programming efforts. The advantage of the approach described in this article is that it involves only commonly used statistical software and so will be a more accessible approach for many statisticians working on clinical trials.

SAS is commonly used among data analysts working on drug trials and is favoured by the pharmaceutical industry in part due to its comprehensive documentation and validation. WinBUGS and OpenBUGS are freely available to download from the website of the MRC Biostatistics Unit in Cambridge where the BUGS project began in 1989 (http://www.mrc-bsu.cam.ac.uk/software/bugs). The BUGS Book is an excellent WinBUGS and OpenBUGS manual and introductory text to Bayesian analysis, written by the group who developed the software.^9^

While BUGS is free, SAS is associated with considerable licence fees and is generally only affordable in large academic departments and pharmaceutical organisations. An obvious alternative to SAS would be to use R Software,^14^ which is free and in some ways a more natural fit given the existing R packages such as R2WinBUGS and BRugs available from CRAN,^15^ the Comprehensive R Archive Network, which incorporate functions for running BUGS through R, and even embedding OpenBUGS in R. The principles outlined in this article would similarly apply to managing the interface between R and WinBUGS when running a simulation study.

Within the context of regulated clinical trials, however, the rigorously validated SAS software is widely used among clinical trial statisticians and is typically preferred over R in the pharmaceutical industry; this article illustrates how to extend statistical programming in SAS to embed Bayesian modelling with BUGS for the development of adaptive clinical trial designs.

## FUNDING

The DexFEM trial is funded by Medical Research Council award MR/J003611/1. The MRC Centre for Reproductive Health is supported by Medical Research Council Centre grant G1002033. C.J.W. and R.A.P. were also supported in this work by NHS Lothian via the Edinburgh Clinical Trials Unit.

## DECLARATION OF CONFLICTING INTERESTS

None declared.

## Supporting information

Data S1. Supporting information

Data S2. Supporting information
